# The Relationship Between Body Composition, Physical Activity, Self-Esteem, and Body Image in Female and Male Adolescents

**DOI:** 10.3390/sports13010011

**Published:** 2025-01-08

**Authors:** Ligia Rusu, Denisa Piele, Eva Ilie, Gheorghe Ionescu, Mihnea Ion Marin, Mihai Robert Rusu, Mirela Lucia Calina

**Affiliations:** Sports Medicine and Physiotherapy Department, University of Craiova, 200585 Craiova, Romania; ligia.rusu@edu.ucv.ro (L.R.); denisa.piele@edu.ucv.ro (D.P.); eva.ilie@edu.ucv.ro (E.I.); gheorghe.ionescu@edu.ucv.ro (G.I.); mihai.rusu@edu.ucv.ro (M.R.R.); mirela.calina@edu.ucv.ro (M.L.C.)

**Keywords:** body composition, self-esteem, adolescents, physical activity

## Abstract

The elements of body composition and their correlation with physical activity, body image, and self-esteem are aspects that require in-depth studies. This link should be seen in the context of the percentage of adipose tissue, which can be modeled via physical activity. The objective of this study is to evaluate the relationships between the parameters that define body composition, self-esteem, body image, and physical activity according to gender. This study included 100 females and 100 males with an average age of 22 years. The evaluation included anthropometric parameters, body composition, self-esteem, physical activity index, and body image perception assessment. The results show that the males exhibited a higher percentage of exceeding the upper limit of normal and average weight; in contrast, there were two times more females exhibiting normal weight than those exceeding the upper limit. Therefore, obesity was higher among males. The average fitness score values were 69.07 for females and 76.53 for males, and the mean fitness was within normal limits. Regarding body image, according to the BSQ, we observed that both groups were not satisfied with and were concerned about their body shape. With respect to the Rosenberg self-esteem scale, the average score for females was 20.27, and for males, it was 19.60; the mean self-esteem value was 66% of the maximum value. In terms of the perceived ideal body size assessed with the Silhouette scale, most of the females were placed at level 3, and the males were placed at level 4. Regarding the physical activity index, females carried out on average 1.5 days of intense physical activity over 7 days, and males were involved in intense physical activity for 2 days. Conclusions: The degree of obesity and therefore the risk of developing cardiovascular disease and metabolic syndrome were higher in males than in females. Although males have a higher degree of obesity, self-esteem is quantified at a higher level of confidence.

## 1. Introduction

Human behavior is generally considered to be multi-dimensional and based on physical appearance and social and educational levels. Health is closely linked to maintaining an active lifestyle; at the same time, we have to manage risk factors that are related to tissue and functional components. In this context, the elements of body composition and their correlation with physical activity are aspects that require in-depth studies, as there are many questions in this regard.

The link between body composition, body mass index, and physical activity can be seen as follows: The percentage of adipose tissue, body mass index, and abdominal circumference are indicators of obesity, which can be modeled via physical activity. Moreover, the analysis of this link in adolescents and young people can also be extrapolated to the way in which this category of people perceives themselves in terms of body image and self-esteem, since adolescence is a period that involves a series of physical, psychological, and social changes associated with changes in body composition.

A cross-sectional study by Joubert [1] focused on the association between body image distortions, lifestyle, and body composition in adolescents. The authors assessed body image using the Silhouette scale, Body Shape Questionnaire, and the Sociocultural Attitudes Towards Appearance Questionnaire-3 (SATAQ-3). Some authors have analyzed the relationship between silhouettes and the percentage of fat mass (adipose tissue calculated via anthropometry) or body mass index [2,3]. The same authors studied body image and its relation to a teenager’s lifestyle, which is very different from person to person. Those who were the most dissatisfied with their body image were sedentary people, and one of the factors negatively correlated with body image was the number of meals per day. The conclusion of this study was that the assessment of lifestyle and body composition requires further research; in addition, further analysis of behavioral risk factors is needed. In many situations, adolescents do not have a sufficient and safe level of physical activity to maintain or improve their health, which has led to this being prioritized as a public health issue.

Weiss and Ebbeck [4], in their paper based on Harter’s competence motivation theory, mention that there are typologies of the relationship between somatic and functional parameters, age, and psychological characteristics. In their study, they discuss a model to understand the motivation for physical activity in adolescents, which illustrates the importance, for self-esteem, of having a certain behavior related to physical activity. Thus, in such a model, the perception of social components and social support is considered to be a determinant of self-perception and obtaining pleasure when engaging in physically active behavior. Self-esteem is important in human behavior, which is multidimensional (physical, social, and academic). Because self-perception is related to physical activity, promoting and developing a positive attitude of self-perception are ways to develop an active lifestyle. Self-esteem is one side of self-perception, and it also has a multidimensional structure, which refers to sports skills, body attractiveness, and physical conditions, elements that are, however, variable according to gender.

In the case of females, the concept is a “thin ideal”, and failure to obtain such a body is associated with a low level of self-esteem. In contrast, males associate self-esteem with the existence of power and strength, and physical activity is an arena in which they can prove their masculinity.

The concept of body image includes an anthropometric assessment, an estimation of attractiveness, and how the person perceives their body shape and size. All this is connected with physiological, cognitive, and socio-cultural parameters. Adolescence is a critical period in the development of body image [5,6], in which physical changes are rapid and diverse. Accepting these changes is essential to the self-perception of body image. Sometimes, these changes may represent risk factors for the formation of a distorted perception of body image. For this reason, it is necessary to have interventions for the way in which an adolescent perceives their body. These interventions are based on objective results that reflect an identification of the factors that generate them, such as biological, psychological, and social factors, age, and health education [7], as well as factors such as body weight control and involvement in physical activity; these all require further analysis.

Studies, such as that of Chae et al. [8], have analyzed the link between BMI and body perception, classified into two categories: underperception and equal perception. In order to increase the accuracy of the information and details related to adolescents’ self-perception, numerous studies which would allow the development of intervention strategies are needed.

Self-perception is considered to be related to physical activity among young people; therefore, the development of a positive self-perception may be important in terms of the approach to physical activity and lifestyle [9].

A complex approach to these issues is related to the correlation between self-perception, self-esteem, and the physical activity performed in relation to fitness components (cardiorespiratory, muscular, flexibility, body composition). Studies have examined the relationship between physical activity, adiposity, cardiorespiratory fitness, and self-perception in adolescents, but the relationship between physical activity, self-perception, self-esteem, and body composition is still unclear [10].

There is international discussion about the so-called “thin ideal”, which seems to start at the age of 9 years. Lubans et al. [11] proposed to analyze gender differences in terms of the link between muscular strength, body composition, and self-perception and to evaluate the subdomains that define self-perception, represented by the link between muscular strength, adiposity, and how people perceive themselves physically. They concluded that there was an inverse relationship between adiposity and physical perception in females, while the relationship between muscle strength and physical perception was directly related in males. The authors mention that a number of studies have tracked the relationship between self-perception, physical activity, cardiorespiratory fitness, and adiposity [12], and muscle strength has been identified as important in determining the health status of adolescents; thus, guidelines have been developed to promote the development of muscle strength in children and adolescents as part of a physical activity program. The importance of physical exercise in determining self-perception is addressed in Lubans et al. [11]; however, this relationship is not fully understood and substantiated. Thus, it was observed that an 8-week exercise program indicated an improvement in anthropometric parameters and self-perception. This means that the observed changes contributed to the shaping and building of self-perception. However, further research is needed to provide evidence of this effect. The study of body image is based on different methods such as interviews, questionnaires, and silhouette perception [13].

Other studies have investigated body image using questionnaires, whose responses were correlated with body composition. Silhouette, as an assessment method, is a form of current body size perception (CBS), which is closely related to body fat.

In recent years, Arsandaux et al. [14] investigated self-esteem associated with factors related to childhood/adolescence or young adulthood in male and female college students. They found that low self-esteem was associated with these factors with special aspects linked to parents’ support and life events, with young adulthood linked with BMI and dissatisfaction with their social life but not physical activity. Their conclusions address many factors related to young adulthood and show more variability among males, whereas the opposite was observed for females.

In 2021, Stagi et.al. [15] published a paper regarding the association between self-perceived body image and body composition in the sexes and in different age classes, using Williamson’s silhouettes to assess the CBS. They determined that these are strongly related to body fat in both sexes until an older age. The whole sample comprised mostly normal weight individuals, including both sexes and classes of age, showing a coherent perception of their current body size.

In terms of the current scientific evidence, there are not many studies that include young people/adolescents in research on the relationship between body composition, CBS, and physical activity in terms of gender differences. Such a study would help to develop a strategy to increase participation in physical activity and to rethink lifestyles.

The aim of our work is to evaluate the relationships between the parameters that define body composition, self-esteem, and body image appraisal through silhouette assessment, the Body Shape Questionnaire, and physical activity, according to gender.

## 2. Materials and Methods

### 2.1. Sample

This study was conducted on 100 females and 100 males, with an average age of 20.9 years, who were students of specialization physiotherapy at the University of Craiova (UCV). All signed provided informed consent, and the research respected the Helsinki Declaration (version 2013) and was approved by the ethics committee of the University of Craiova (UCV) (Ref 254/12.12.2023).

The selection of subjects was based on the following inclusion criteria:

- Young people aged 19–25,
- No chronic diseases—diabetes, metabolic syndrome,
- Ability to carry out daily activities.

The exclusion criteria were as follows:

- Neuropsychic and locomotor disorders,
- Constant sports activity (practicing a regular sport activity, training for a specific sport),
- Adoption of diets.

To recruit the research group, we used sampling techniques based on simple random sampling. No participants withdrew from the research, and we analyzed the data from all the participants.

### 2.2. Study Design and Instruments

This study was conducted from May to June 2024 and included the evaluation of subjects from an anthropometric point of view (height, weight, and body mass index), body composition assessment, self-esteem assessment, physical activity index assessment, and body image perception assessment (Table 1).

The anthropometric assessment was based on height measurements, using a height meter (stadiometer), as well as weight, body mass index, and body composition, assessed using the InBody 230 equipment. InBody 230 utilizes bioelectrical impedance analysis (BIA) technology to measure human body composition. The subjects were assessed under basal conditions, in the morning, under conditions of a 12 h fasting period, with adequate hydration, without having performed intense physical activity for 24 h prior to the assessment. The parameters that were extracted and analyzed were the target weight, fitness score, skeletal muscle mass, body fat mass, and abdominal obesity degree. In the case of the abdominal obesity degree, there were reference values assigned by the InBody 230 equipment, against which we reported our results.

The evaluation of self-esteem was performed using the Rosenberg self-esteem scale (RSES) [16], which includes 10 items consisting of statements addressing general feelings about oneself. The participants received a score for each of the 10 items by selecting one of the numbers 0, 1, 2, or 3, and the total score was calculated by summing the partial scores. Thus, the minimum total score is 0, and the maximum is 30.

Body image perception was assessed using the Body Shape Questionnaire (BSQ-16B) [17], which includes 16 items and reveals feelings about appearance over the last four weeks. For this questionnaire, participants received a score for each of the 16 items by selecting numbers from 1 to 6, and the total score was calculated by summing the partial scores. The minimum score is 16, and the maximum is 96.

Additionally, body shape perception was evaluated using the Silhouette scale, which assesses perceived ideal body size [18]. For this questionnaire, each participant selected one of the 9 items. The items were ranked from 1 to 9, and the final score was determined by the position of the selected item in the hierarchy. The minimum score is 1, and the maximum is 9.

The level of physical activity was assessed using the Index of Physical Activity from the International Physical Activity Questionnaires (IPAQ) short form, which includes daily activities [19]. For this questionnaire, each participant recorded the number of minutes per day (IPAQ 2, 4, 6, and 7) or the number of days per week (IPAQ 1, 3, and 5) spent on activities associated with each of the 7 questions. The minimum value for all questions is zero, while the maximum value for IPAQ 1, 3, and 5 is 7.

The assessment protocol collected the personal information (date of birth, previous pathology, sport activity), measured the body composition, and applied the scales: Rosenberg, BSQ-16B, Silhouette, and IPAQ. All data were stored in a folder created for each participant. Statistical analysis was applied to all the data. The evaluations were conducted by the researcher according to GDPR rules. The next steps included measurement of height, conceptualization by a manager, and measurement and evaluation by assistant managers, who are physiotherapists. We conducted one assessment session for each subject, lasting 45 min.

### 2.3. Statistical Procedure

Differences in the average values of numerical indicators were evaluated using the independent samples *t*-test with a level of significance alpha = 0.05. Pearson correlation was used to describe the associations between all selected variables. The statistics used included descriptive statistics of the recorded results, Pearson correlations, and inferential statistics, all using XLStat software [20].

## 3. Results

### 3.1. Anthropometric and Body Composition Assessment

Table 2 shows the mean values of the anthropometric characteristics for the two groups of subjects.

Analyzing the data, the following observations were made:

For the females,

- The central weight (50% of values) varied within a relatively narrow range (interquartile range is 4.62 kg), indicating a homogeneous distribution;
- They had relatively similar heights within the interquartile range;
- The BMI values were similar in the median zone, showing low variations, and the mean was 22.79.

For the males,

- There was a large dispersion (interquartile range is 15.1 kg) in the central weight, indicating significant variations in constitution;
- The heights in the median range showed greater variation compared to females;
- BMI exhibited moderate variation, and the mean was 25.47.

Table 3 shows the data records reflecting body composition, which express the ideal body weight values, fitness score, skeletal muscle and fat mass, and the degree of obesity via the abdominal obesity index.

Within the interquartile range, females showed small variations in muscle mass, but the body fat presented significant variations, while within the interquartile range, males’ muscle mass and body fat varied moderately.

The standard values of the parameter, percent body fat, which is a representation of the fat tissue mass, were those specified in the InBody equipment manual; the average value of the percentage of adipose tissue was 15% in males and 23% in females, and the range considered normal is 10–20% in males and 18–28% in females:

- In total, 43% of males were below the threshold value of 15%; the rest were above this threshold value;
- In total, 77% of females were below the threshold value of 23%; the rest were above this threshold value;

If we relate the results to the range considered normal, the distribution of subjects was as follows:

- In total, 43.4% of males fell in the 10–20% range considered normal, 16.6% were below the 10% body fat threshold, and 40% were above the 20% body fat threshold.
- In total, 40% of females fell in the 18–28% range considered normal, 43.3% were below the 18% body fat threshold, and 16.7% were above the 28% body fat threshold.

Analysis of these results shows that in males, there was a high percentage who exceeded the upper limit of normal and average, unlike females, in which the normal group was two times larger than those who exceeded the upper limit. Obesity was therefore higher among males.

Regarding the quantification of abdominal obesity,

- In total, 66% of males were below the threshold value of 90%, with the rest above this threshold value;
- In total, 66% of the females were below the 85% threshold value, with the rest above it.

The fitness score values indicated average values of 69.07 for females and 76.53 for males; hence, these values corresponded to a state of health and a level of fitness within normal limits.

### 3.2. Assessment of Body Image Perception, Self-Esteem, and a Scale Used to Assess Perceived Ideal Body Size

Table 4 and Table 5 show the results of the application of the three questionnaires (BSQ, Rosenberg, and Silhouette scale) for the two groups of subjects.

With regard to the interquartile range, the following remarks can be made:

For the females,

- The BSQ scores were highly dispersed, indicating varied body perceptions.
- The Rosenberg scores were relatively homogeneous in the median range.
- The SHIL scores showed small variations.

For the males,

- They had less dispersion in body perception scores than the females.
- The Rosenberg scores varied more compared to females.
- The SHIL scores were similar.

It can be observed, according to the BSQ questionnaire, that both females and males were not satisfied with and were concerned about their body shape; the average values were lower than 80 points, and averages for both groups were close.

According to the results of the Rosenberg self-esteem scale [16], the average score for females was 20.27, and for males, it was 19.60. In relation to the evaluation range, 0–30, we found that both groups showed self-esteem at 66% of the maximum value. Analyzing the maximum values, males were more confident, with a self-esteem close to the maximum value.

In terms of the perceived ideal body size assessed with the Silhouette scale [18], most of the females were at level 3, with most of the males at level 4.

On this issue, the position of the subjects was as follows:

- In total, 13.3% of males were between 2 and 3,
- Hence, 86.7% were between 4 and 7.

A higher percentage of males perceived themselves to have a silhouette that corresponded to a pre-obese or obese state. In total, 53.3% of females were categorized between 1 and 3 and 46.7% between 4 and 6, which means that the females had a perception that corresponded to a slim body shape.

These aspects are in some agreement with the percentages represented by those found in both males and females in relation to the mean values of the percentage of fat (15% in males and 23% in females), because there was a predominance of females below the mean threshold value and a predominance of males above the mean threshold value.

### 3.3. International Physical Activity Index (IPAQ)

Regarding the physical activity index assessed using the IPAQ scale, the results are presented in Table 6 and Table 7, corresponding to the two groups of subjects. In the interquartile range, the females exhibited a wide range of physical activity levels, while the males exhibited less variation.

It can be observed that females achieved on average 1 day and a half of intense physical activity over 7 days, where males were involved in intense physical activity for 2 days. As for the duration of intense physical activity, again females had one hour or less, while males exceeded one hour per day. As for moderate activity, the average number of days per week was higher for males than for females, and as for the duration per day, females performed moderate physical activity for a longer period than males. Walking was an activity that both females and males stated they performed on average 6 days per week, and as for the number of hours per day, again females spent more time. IPAQ items 3, 4, 5, and 6 indicate that females were more involved in moderate physical activity than males. Regarding the preference for adopting the sitting position, this was higher in males than in females, with males preferring this position 1 h more than females, daily. Therefore, these data reveal females have a higher involvement in physical activity than males.

Females tended to have less dispersion in physical parameters (e.g., weight, BMI) but greater variation in body perception scores (BSQ).

Males exhibited greater dispersion in weight and height but had more uniform psychosocial scores.

### 3.4. Statistical Analysis of Results

For the statistical analysis, we performed the Student *t*-test, with a level of significance alpha = 0.05, which allowed us to quantify the differences between the two groups of subjects, males and females.

#### 3.4.1. Statistical Analysis of Body Composition Parameters

A statistical analysis of the body composition parameters was made using the Student *t*-test (alpha = 0.05), which showed that there were significant statistical differences between females and males for five out of the six parameters, as shown in Table 8.

There was a significant statistical difference in the target weight, fitness score, skeletal muscle mass, abdominal obesity degree, and body mass index.

#### 3.4.2. Statistical Analysis of the Questionnaires

A statistical analysis of the parameters for the BSQ and Rosenberg was conducted using the Student *t*-test (alpha = 0.05), which showed that there were significant statistical differences between females and males, as shown in Table 8.

Concerning the IPAQ physical activity index questionnaire, the *t*-test applied to the IPAQ questionnaires showed a significant difference between females and males for IPAQ item 3 (alpha = 0.005), Table 8.

**Figure 1:**
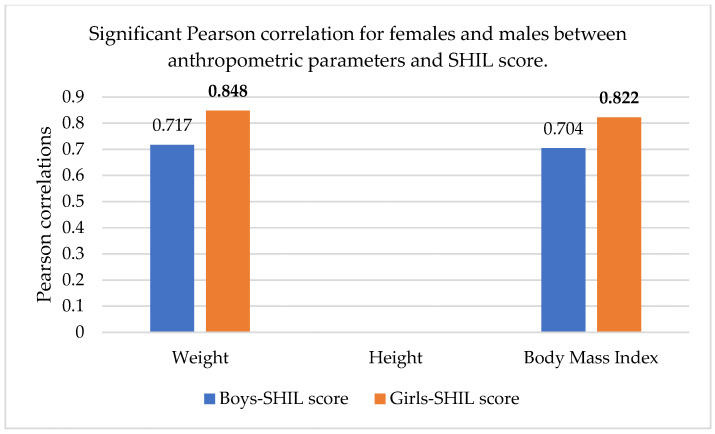
Significant Pearson correlation for females and males between the anthropometric parameters and SHIL score. The bold values are different from 0 with a significance level alpha = 0.05.

As part of the statistical analysis, we also performed correlations between the measured parameters; the correlations are shown in Table 9, using Pearson correlation (Figure 1).

There was a much stronger correlation for females than males, regarding the two parameters of body weight and BMI, on the one hand, and the SHIL score, on the other hand, which means that there was indeed a stronger link between body weight, the care for its maintenance, and the way females perceived themselves.

**Figure 2:**
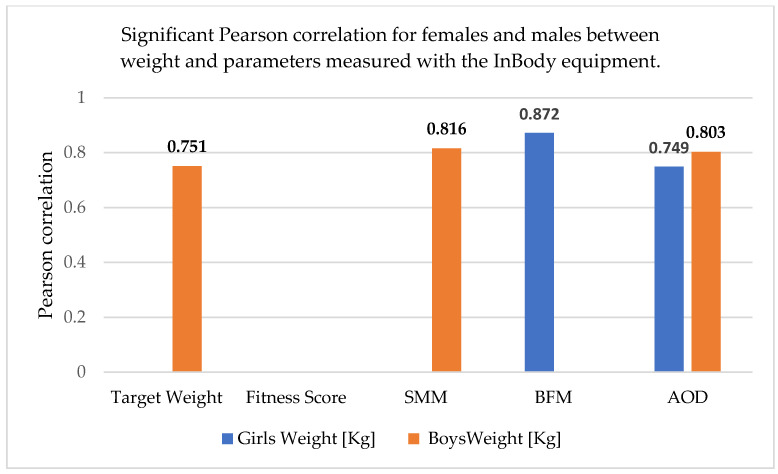
Significant Pearson correlation for females and males between weight and parameters measured with the InBody equipment. The bold values are different from 0 with a significance level alpha = 0.05.

**Figure 3:**
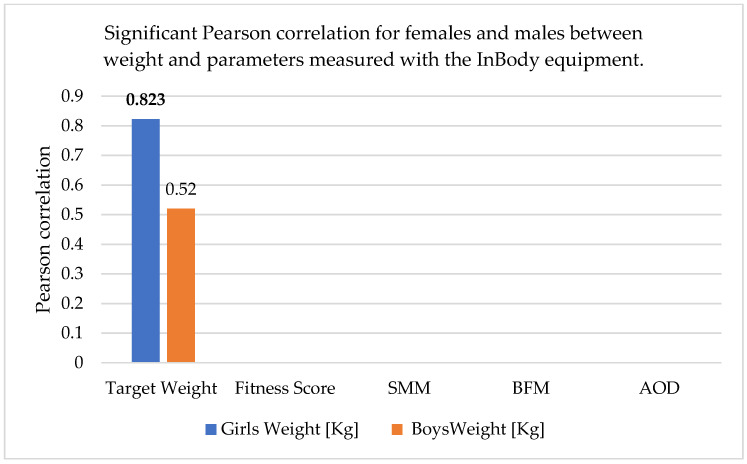
Significant Pearson correlation for females and males between height and parameters measured with the InBody equipment. The bold values are different from 0 with a significance level alpha = 0.05.

**Figure 4:**
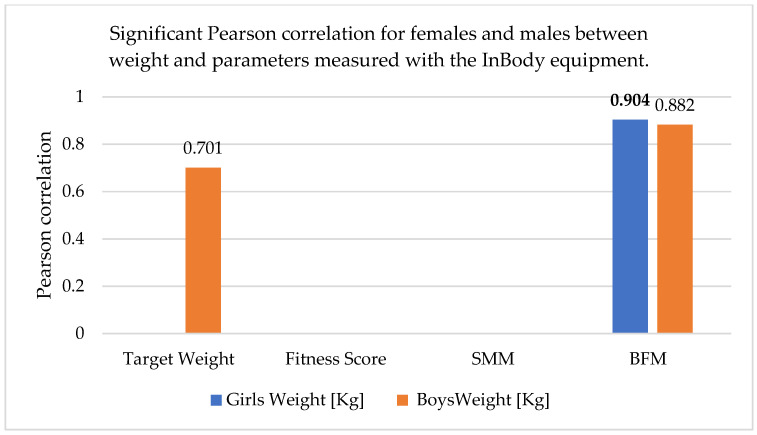
Significant Pearson correlation for females and males between body mass index (BMI) and parameters measured with the InBody equipment. The bold values are different from 0 with a significance level alpha = 0.05.

The correlations realized between the anthropometric parameters and body composition parameters obtained with the InBody equipment are shown in Table 10 and Figure 2, Figure 3 and Figure 4 (skeletal muscle mass = SMM, body fat mass = BFM, abdominal obesity degree = AOD).

In females, there was a better correlation between weight and height and between adipose tissue mass and weight. In males, there was a relatively good correlation between skeletal muscle mass and weight.

Therefore, it appears that the representativeness of adipose tissue and skeletal muscle mass as correlated with weight is gender-specific. As can be seen, the fitness score did not correlate with any of the parameters, and the abdominal obesity degree was correlated with weight and BMI in both males and females, but the correlation was stronger in males.

We also performed a correlation analysis between the body composition parameters and the scores resulting from the application of the BSQ, Rosenberg, SHIL, and IPAQ questionnaires. The results show that for females, Pearson correlations existed only between the SHIL score and weight (r = 0.848), body mass index (r = 0.822), and target weight (r = 0.702). For males, correlations were also observed between the SHIL score and weight (r = 0.717), as well as body mass index (r = 0.704). No correlations were found between the other parameters.

**Figure 5:**
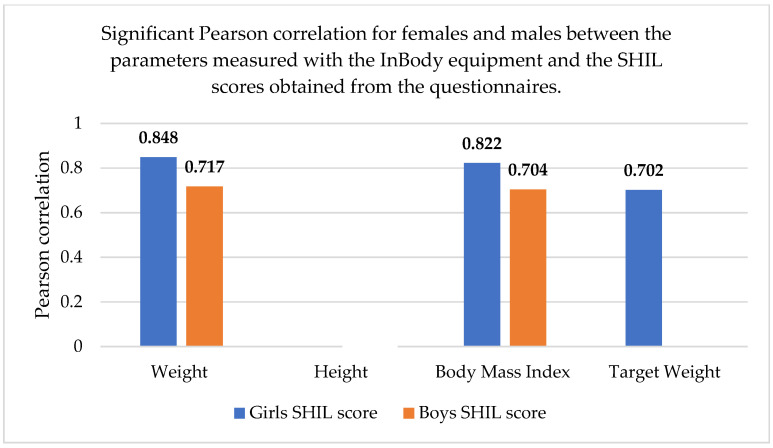
Significant Pearson correlation for females and males between parameters measured with the InBody equipment and the SHIL scores. The bold values are different from 0 with a significance level alpha = 0.05.

The graphic representation of the correlations between the body composition parameters and the scores resulting from the questionnaires is presented in Figure 5.

We observe that there were no strong correlations between these parameters, and as we mentioned earlier, the only possible correlations were between the way subjects perceived themselves, their actual weight, and implicitly, their body mass index.

From the calculation of Pearson correlations between the scores resulting from the application of the BSQ, Rosenberg, SHIL, and IPAQ questionnaires in both groups of analyzed subjects, the following results were obtained:

- For females, a weak correlation was found only between the IPAQ 1 and IPAQ 4 scores (r = 0.589).
- For males, a weak correlation was found only between the IPAQ 1 and IPAQ 2 scores (r = 0.657), with a weak inverse correlation between the SHIL score and IPAQ 4 (r = −0.531).

## 4. Discussion

Our study highlights the fact that there are important differences between females and males in terms of body weight and body fat percentage; if we refer to the reference values, there are significant differences in body composition, as also mentioned in Petrekova [21], which specified that there were differences in body composition between men and women and in physical activity, with females being more involved in moderate physical activity than males. Regarding how they perceive themselves and how they are concerned about body image, females also show interest in and evaluate their body image more correctly, related to the SHIL scale, closely related to weight, as found in the study of Chang et al. [22], with 90% of the students involved in the study. If we refer to the BSQ questionnaire on body shape, which showed a lack of concern in terms of how they appreciate their body shape, it is observed that this is also reflected in self-esteem, which was evaluated by the Rosenberg scale. This showed that although both groups of subjects had average values within the reference values, when we analyzed the relationship with the maximum value, we found that self-esteem was 66% of the maximum value, with a higher degree in males than in females. This aspect agreed with the results reported by Aggarwal [23]. A positive body image was linked to increased confidence and wellbeing, whereas a negative body image was linked to lower self-esteem.

In terms of our results regarding the relationship between the SHIL and body weight, body fat percentage, and BMI, there was a correlation, which was also reflected in the work of Crolini et al. [24], who concluded that the current body size was correlated with BMI both in males and in females, with a strong concurrent validity and a large effect in both samples [25]. In addition, the correlation coefficient between the current body size measured by our scale and BMI in the second sample was similar to the one between the current body size measured by the Thompson’s scale and BMI, with a large effect.

The same aspect of the correlation between SHIL and body composition, more specifically weight and BMI, can be found in Stagi et al. [15], who conducted a study on the relationship between self-perceived body image and body composition and found that it was positively correlated with weight and BMI in both groups.

Regarding the perception of body shape, our results, based on the BSQ questionnaire, did not reveal differences between males and females, but in terms of correlations, the closest relationship seems to be with abdominal obesity degree and not with body fat mass, in the sense that the relationship is much smaller in females (r = 0.230) than in males (r = 0.330), an aspect also found in the article by Kagawa et al. [26].

Related to physical activity, we observed that there was no correlation between the IPAQ scores and body composition items, which is in agreement with the observations of Wu et al. [27], who mentioned that no significant difference in the waist-to-hip ratio was found in their study after the eight-week intervention. They underlined the same aspect as we found regarding a link between abdominal obesity degrees and weight, with respect to body fat mass. Their study showed that abdominal-fat weight was positively correlated with BMI, body fat weight, and body fat percentage.

What is surprising is the result reflecting self-esteem, which correlated negatively with all the measured parameters. In addition, the fitness score is a parameter that indicates a good level of fitness and correlates somewhat with performing an intense daily physical activity, which had an average value of 1–2 days per week. The analysis of the fitness score in relation to the anthropometric parameters did not show a correlation. We also did not find a correlation with the degree of involvement in physical activity, assessed by the IPAQ scale; this aspect is also mentioned by Gilani et al. [28], who analyzed the relationship between physical activity, BMI, and body composition and concluded that physical activity had a weak negative and weak positive correlation with fat mass and muscle mass, respectively. In addition, there was a very weak negative correlation between physical activity and BMI. Nevertheless, there was a significant relationship between physical activity and body composition, i.e., fat mass and muscle mass. Meanwhile, physical activity had no significant relationship with BMI. The existence of a normal fitness score in both groups, associated with moderate and intense physical activity by males, daily, is an aspect that we find also in the literature. The results showed that the mean SE scores increased significantly in participants who received the intervention. Participating in regular aerobic exercise for eight weeks as an intervention appeared to increase the mean SE score [28].

As with Zogg et al. [29], we observed an inverse correlation, although insignificant, between the IPAQ and body fat mass. The authors mentioned that, in multiple linear regressions, leisure-time activity showed an inverse association with waist circumference and relative fat mass and a positive correlation with relative muscle mass.

Our study has allowed us to identify parameters that can differentiate the behavior of the body composition–self-esteem–body image relationship, depending on gender.

Analyzing some studies regarding body composition, we found that Kirchengast et al. [30] demonstrated that females, even with a normal weight, had a significant percentage of relative fat mass, whereas normal weight males showed a significantly higher amount of fat-free body mass. In the same study, the authors examined the level of physical activity and found that males were significantly more physically active than females.

Regarding other aspects, such as body image, self-esteem, and body perception, Russo et al. [31] found a dissatisfaction with body image, as we did. In addition, the authors discussed the sports activity that was considered to have a determining factor in the perception of the silhouette. According to this study, we used the SHIL scale, but we did not report the relationship between the attractiveness to the opposite sex, as the authors did. They found that the FAD index was based on the difference between the silhouette perceived as one’s own real body image and the silhouette of the same sex considered to be attractive to the opposite sex.

In terms of physical activity, Slavica [32] proposed a physical activity score, and their investigation showed that a game was a significant predictor of differences in physical activity between males and females, and that males played more games than females.

## 5. Conclusions

The degree of obesity and, therefore, the risk of developing cardiovascular disease and metabolic syndrome is higher in males than in females.

In examining how males and females perceive their body shape according to the BSQ scale, both groups, although not satisfied, were not concerned about this aspect.

Although males have a higher degree of obesity, their self-esteem showed a higher level of confidence. In accordance with the degree of obesity, males perceived their own body image, at a pre-obesity level, although in terms of the abdominal obesity degree, most males were below the threshold value of 90%.

In terms of physical activity as measured by the IPAQ scale, males engaged predominantly in intense physical activity, while females sustained moderate physical activity but for a longer duration. In addition, because the participants were students from a physiotherapy course, it is possible there was a difference regarding physical activity according to their physical preparation, as they can be included in physical activity more than other people.

Limitations of this study—the participants came from both rural and urban backgrounds, which may result in different dietary habits and lifestyles, impacting the results of our study. The subjectivity of responses may also influence the results of this study. This aspect would not influence body composition but could affect self-esteem. 

Further, as the participants were not randomly selected from the wider population, there may have been volunteer bias.

## Figures and Tables

**Table 1:** Stage of activities.

ITEMS	May 2024	June 2024
Recruitment of the participants	x	
Assessment processes	x	x
Data collection	x	x
Data analysis		

**Table 2:** Values of anthropometric parameters.

Parameters	Females				Males			
	Age [years]	Weight [Kg]	Height [cm]	BMI [Kg/m^2^]	Age [years]	Weight [Kg]	Height [cm]	BMI [Kg/m^2^]
Minimum	19.00	45.80	155.00	17.20	19.00	53.40	162.00	16.10
Maximum	23.00	91.70	172.00	34.50	25.00	111.00	190.00	33.10
1st Quart.	20.00	52.63	160.25	19.05	20.25	67.13	173.00	22.25
Median	21.00	57.25	163.00	21.90	21.00	82.30	181.00	25.30
3rd Quart.	21.00	66.78	167.00	24.80	21.75	92.25	183.75	28.45
Mean	20.90	60.91	163.53	22.79	21.20	81.03	178.87	25.47
Std.dev.	0.96	11.80	4.61	4.40	1.27	16.68	7.74	4.60

**Table 3:** Body composition parameter values.

**Parameters**	**Females**					**Males**				
	**Target Weight [Kg]**	**Fitness Score**	**Skeletal Muscle Mass [Kg]**	**Body Fat Mass [Kg]**	**Abd. Obesity Degree**	**Target Weight [Kg]**	**Fitness Score**	**Skeletal Muscle Mass [Kg]**	**Body Fat Mass [Kg]**	**Abd. Obesity Degree**
Min	51.60	46.00	15.90	8.10	0.64	58.50	59.00	22.50	5.50	0.76
Max	68.10	87.00	30.90	39.20	1.06	92.30	93.00	45.40	40.50	0.95
1st Quart	55.18	66.00	19.73	11.73	0.79	72.15	70.50	33.30	12.95	0.84
Median	58.90	70.50	22.40	19.90	0.81	76.75	78.00	36.80	15.35	0.87
3rd Quart	61.23	72.00	24.88	22.45	0.86	84.90	80.00	42.18	23.85	0.91
Mean	58.63	69.07	22.54	19.63	0.83	77.75	76.53	36.65	18.01	0.87
Std.dev	4.15	7.10	3.63	8.58	0.08	9.72	8.64	6.56	8.50	0.05

**Table 4:** Results of the BSQ, Rosenberg, and Silhouette for the female assessments.

	BSQ Total	Rosenberg Total	SHIL Score
Minimum	16	13	1
Maximum	61	26	6
1st Quartile	26.25	19	3
Median	38.50	20	3
3rd Quartile	48.75	22.75	4
Mean	37.40	20.27	3.53
Std.dev	13.22	3.19	1.31

**Table 5:** Results of the BSQ, Rosenberg, and Silhouette for the male assessments.

	BSQ Total	Rosenberg Total	SHIL Score
Minimum	18	11	2
Maximum	64	29	7
1st Quartile	24.50	17	4
Median	32	19	4
3rd Quartile	37	22	5
Mean	32.37	19.60	4.37
Std. dev	10.66	3.77	1.13

**Table 6:** IPAQ—females’ values.

	IPAQ1 days/week	IPAQ2 min/day	IPAQ3 days/week	IPAQ4 min/day	IPAQ5 days/week	IPAQ6 min/day	IPAQ7 min/day
Min.	0	0	0	0	3.00	60	120
Max.	7	300	7.00	600	7.00	600	600
1st Quart	0	0	0.25	60	7.00	120	240
Median	1	60	2.00	120	7.00	120	300
3rd Quart	2	120	4.00	180	7.00	180	360
Mean	1.73	66	2.57	160	6.57	162	298
Std.dev	2.26	72.81	2.08	158.87	1.04	101.08	108.58

**Table 7:** IPAQ—males’ values.

	IPAQ1 days/week	IPAQ2 min/day	IPAQ3 days/week	IPAQ4 min/day	IPAQ5 days/week	IPAQ6 min/day	IPAQ7 min/day
Min.	0	0	1	15	2	30	60
Max.	5	180	7	480	7	480	720
1st Quart	1	30	3	75	7	60	180
Median	3	120	4	150	7	120	240
3rd Quart	3	120	5	180	7	120	420
Mean	2.40	87	3.87	150.50	6.57	120	336
Std.dev	1.73	56.46	1.68	94.42	1.22	95.19	212.34

**Table 8:** Statistical results of the body composition parameters and the BSQ, Rosenberg, Silhouette, and IPAQ scores.

Parameters	Difference	*p* *
Target Weight [Kg]	−19.12	**0.001**
Fitness Score	−7.47	**0.001**
Skeletal Muscle Mass [Kg]	−14.11	**0.001**
Body Fat Mass	1.62	ns
Abdominal Obesity Degree	−0.04	ns
Body Mass Index [Kgm^2^]	−2.68	ns
BSQ TOTAL	5.03	ns
Rosenberg TOTAL	0.67	ns
SHIL Score	−0.83	**0.026**
IPAQ1 days/week	−0.667	ns
IPAQ2 h/day	−21	ns
IPAQ3 days/week	−1.3	**0.014**
IPAQ4 min/day	−10.5	ns
IPAQ5 days/week	0	ns
IPAQ6 min/day	42	ns
IPAQ7 min/day	−38	ns

* *p*-value two-tailed. The bold values are different from 0 with a significance level alpha = 0.05. ns—not significant with level alpha = 0.05.

**Table 9:** Pearson correlation for females and males between anthropometric parameters and SHIL score.

Variables	Females: SHIL Score	Males: SHIL Score
Weight	**0.848**	**0.717**
Height	0.083	0.160
Body Mass Index	**0.822**	**0.704**

The bold values are different from 0 with a significance level alpha = 0.05.

**Table 10:** Pearson correlation for females between the anthropometric parameters and the parameters measured with the InBody equipment.

Parameters	Females			Males		
	Weight [Kg]	Height [cm]	BMI [Kg/m^2^]	Weight [Kg]	Height [cm]	BMI [Kg/m^2^]
Target Weight	0.532	**0.823**	0.292	**0.751**	0.520	**0.701**
Fitness Score	−0.259	0.079	−0.297	0.302	0.066	0.339
SMM	0.691	0.443	0.558	**0.816**	0.454	0.793
BFM	**0.872**	−0.114	**0.904**	0.761	0.092	**0.882**
AOD	**0.749**	−0.306	**0.845**	**0.803**	0.057	**0.960**

The bold values are different from 0 with a significance level alpha = 0.05.

## Data Availability

The data that support the findings of this study are available from the corresponding author upon reasonable request.
